# Nervous Diseases

**Published:** 1878-11

**Authors:** 


*Nervous Diseases: Their Description and Treatment.* By Allan McLane, Hamilton, M. D., etc., etc. With Fifty-Three Illustrations. Philadelphia: Henry C. Lea. 1878. Buffalo: Theodore Butler, 307 Main Street.

The author tells us that it has been his aim "to produce a concise, practical book," and in this he has met with a very flattering success. His work, while giving thoughtful attention to matters of diagnosis and treatment, at the same time does not fail to take a comprehensive view of the etiology and pathology of nervous affections. Aside from the introduction which treats of the methods of diagnosis and the instruments for diagnosis and treatment, the book is divided into eighteen chapters which are respectively, as follows: Chapter I, Diseases of the Cerebral Meninges; Chapters II to VII, Diseases of the Cerebrum and Cerebellum; Chapter VII, Diseases of the Spinal Meninges; Chapters VIII to XI, inclusive, Diseases of the Spinal Cord; Chapter XII, Bulbar Diseases; Chapters XIII and XIV, Cerebro-Spinal Diseases; Chapters XV to XVIII, Diseases of the Peripheral Nerves. A few pages at the end are occupied by Formulæ.

The introduction contains some valuable instruction upon the methods of examination of patients, and also gives some very concise directions concerning post mortems in cases of nervous disease. This is a subject upon which general practitioners are too often deficient in information, which is only to be obtained, as a rule, in works upon morbid anatomy and pathology unattainable by the majority of physicians. In speaking of the ophthalmoscope in diagnosing cerebral lesions we are glad to observe that Dr. Hamilton has the bravery and honesty to place a just estimate upon its utter inutility in diagnosing disturbances of the intra-cranial circulation. The author considers Seguins' surface thermometer amply sufficient to detect variations in temperature in various parts of the body. The differential thermo-electric calorimeter of Sombard and other complicated and expensive instruments are speken of as practically useless except for experimental purposes. We wish the author had given us his opinion of the value of Lombard's or any other instrument in detecting cerebral congestion by differences in temperature on the exterior of the cranium. The dynamometer is described, and one of the author's inventions figured, the dynamograph and the myograph are also described. The chapter on Diseases of the Membranes is a careful and intelligent consideration of the subject of which the article on hæmatoma of the Dura-Mater is not the least interesting. No mention is made of sarcoma of the Dura-Mater. Cerebral hyperæmia is well treated but its importance or frequency is not exaggerated as by some other authors, the same may be said also of cerebral anæmia. Nearly thirty pages are devoted to cerebral hemorrhage. On page 93 the author endorses the view of Gower and others, when he says: "Neither clinical history nor supposed pathology of athetosis affords ground for separating it from other forms of disordered movement commonly seen after hemiplegia, but any one of which might occur in the primary affections." The tendency is already too great to invent new names and classifications in nervous diseases, and athetosis must receive more support to its claims as a distinct disease than it has thus far to be received by the profession as such. Strange to say, however, several writers have followed in the track of Dr. Hammond in describing the well known occurrence of disordered and uncontrolled movement of the fingers and toes, as a new disease. An English writer in speaking of these reported cases well says that the disease, inability to control the fingers, seems to have taken possession of the fingers of a certain number of medical writers. The article on Epilepsy is good and very properly takes strong ground against the routine treatment into which many practitioners have fallen. Hysteria is well treated. The article on hystero epilepsy has already been published in the *American Journal of Medical Science.* Diseases of the peripheral nerves are satisfactorily considered. The formulæ which complete the volume are in the main well selected. The illustrations which adorn the volume are judiciously selected and well executed.

We regret that in too many instances the author's style is marred by evidences of hasty writing, and a few inelegant expressions have crept into the text. There are also a few noticeable typographical errors. A "stiff dose" does not sound well, and, although the word is used by an eminent New York surgical writer, we do not like the expression "contractured." On the whole however this work is a valuable one, especially as it does not mislead the reader by given results attained or attainable by no one but the author.

